# Commentary: Does the Coronavirus (COVID-19) Pandemic Call for a New Model of Older People Care?

**DOI:** 10.3389/fpubh.2020.581836

**Published:** 2020-11-02

**Authors:** Tony Kuo, Laura Trejo

**Affiliations:** ^1^Department of Family Medicine, David Geffen School of Medicine at UCLA, Los Angeles, CA, United States; ^2^Department of Epidemiology, UCLA Fielding School of Public Health, Los Angeles, CA, United States; ^3^Population Health Program, UCLA Clinical and Translational Science Institute, Los Angeles, CA, United States; ^4^Department of Aging, City of Los Angeles, Los Angeles, CA, United States

**Keywords:** COVID-19, older people care, aging services, healthcare system, public health infrastructure

## Introduction

In their recent *Frontiers in Public Health* article, Palombi et al. (1) elegantly described the fragility of public health services around the globe and characterized gaps in epidemic preparedness, in particular those within the older people care system that have limited formal and informal support networks. The authors insightfully pointed out that successful public health interventions against coronavirus disease 2019 (COVID-19) such as social distancing paradoxically exacerbate social isolation, a survival risk factor for frail elderly, especially in regions where the proportion of single residents who are over the age of 80 is high. Support for older adults feeds into a system that is often fragmented, with the services' locus of control distributed across multiple sectors. Although the authors went on to suggest possible solutions to addressing these gaps—e.g., use of telemedicine, assistance for specific needs (nutrition and drug supply), disability support, detection of danger signals, and timely prevention and communication—the authors fell somewhat short in suggesting larger policies or recommending multi-sector collaboration that can lead to meaningful system-level changes. Among them would be increasing investments in priority areas such as workforce development or a consensus framework that can be used to help implement effective aging in place interventions in the community (2, 3).

## Challenges to Improving Older People Care

Historically, social and financial investments in community care for older people have been constrained, lacking prioritization among civic leaders and decision-makers. Building a new model of older people care as suggested by the authors is worthwhile but may require looking at solutions or experiences from the past to help guide this effort (3–5). For example, social interventions at the community level using home health care or supportive resources as alternatives to nursing home or congregate living placement are not novel and are generally well-accepted by health professionals because they do not significantly compromise care quality. In spite of these beneficial characteristics, investments in these interventions have remained limited.

Another challenge to a more robust older people care system has been the traditional boundaries of social, health, and public health disciplines. Social services (e.g., social workers, program implementers), health care (physicians, nurses, other health care workers), and public health professionals often do not work together on older adult issues in a coordinated, interdisciplinary way. For instance, the aging services sector, which includes agencies, programs, and activities that support vulnerable older people in the community (6), does not always have easy access to experienced medical advisors within its immediate work environments. Similarly, health and public health sectors do not generally include social work or gerontology experts in their leadership circles. Consequently, decisions about health and public health services delivery are often made without a gerontological lens.

Finally, a shortage of professionals who are prepared to care for older adults has further stressed the older people system in many countries. Lack of prestige in pursuing a career in aging, aging services jobs that are typically low paying, and limited financial incentive programs to recruit and retain top talents in this field have all contributed to a workforce shortage problem. Despite the existence of various policies and laws to support the education and professional development of this workforce, competing interests and priorities have continued to dilute longer term funding for these endeavors (3–5). COVID-19 may have further exposed this need for a better prepared workforce but the pandemic certainly did not create it.

## Age-Friendly Cities and Communities Movement

Multi-sector collaboration could lead the way to making the necessary system changes required to build the new model envisioned by Palombi et al. (1). Although still early in its planning, numerous communities are beginning to form innovative partnerships to prepare for an aging population, basing their efforts on the World Health Organization's Global Network for Age-Friendly Cities and Communities framework (7). Los Angeles, California, USA is one such example. Its *Purposeful Aging Los Angeles* initiative (PALA) (8) brings together regional governments, health agencies, cities, aging advocates (e.g., AARP), the private sector, and universities to collectively plan for an aging population. The regional initiative focuses on facilitating recognized best practices such as the Los Angeles Alliance for Community Health and Aging, a learning collaborative of community services providers that fosters public health and aging sector teamwork “to identify needs and challenges, coordinate supports and services, and leverage funding and other resources [to] best serve the health and social needs of LA's older adults” (5). PALA is seen as the backbone infrastructure for older people care in Los Angeles and offers a vision for how older people care can be improved in the USA, with contributions from such sectors as housing, transportation, health services, commerce, and community support services.

## Discussion

We appreciate the insights and lessons learned shared by Palombi et al. (1). They resonate deeply with many who are working tirelessly to prevent COVID-19 from devastating older populations with high risk comorbidities (9) in their countries. This call to action for a new model of older people care is refreshing and should be operationalized and integrated urgently as part of the response to this pandemic, especially as various countries reopen and move through the different stages of containment, mitigation, and suppression of COVID-19 (10).

## Author Contributions

TK and LT conceptualized and wrote this General Commentary. Both have reviewed and approved this article for publication.

## Conflict of Interest

The authors declare that the project was conducted in the absence of any commercial or financial relationships that could be construed as a potential conflict of interest.
